# Deletion of the Na/HCO_3_ Transporter NBCn1 Protects Hippocampal Neurons from NMDA-induced Seizures and Neurotoxicity in Mice

**DOI:** 10.1038/s41598-019-52413-0

**Published:** 2019-11-05

**Authors:** Hae Jeong Park, Carlos E. Gonzalez-Islas, Yunhee Kang, Jun Ming Li, Inyeong Choi

**Affiliations:** 1 Department of Pharmacology, Kyung Hee University School of Medicine, Seoul, South Korea; 2 Doctorado en Ciencias Biologicas, Universidad Autonoma de Tlaxcala, Tlax, Mexico; 3 Department of Physiology, Emory University School of Medicine, Atlanta, GA 30322 USA; 4 Department of Human Genetics, Emory University School of Medicine, Atlanta, GA 30322 USA

**Keywords:** Diseases, Cell death in the nervous system

## Abstract

The Na/HCO_3_ cotransporter NBCn1/SLC4A7 can affect glutamate neurotoxicity in primary cultures of rat hippocampal neurons. Here, we examined NMDA-induced neurotoxicity in NBCn1 knockout mice to determine whether a similar effect also occurs in the mouse brain. In primary cultures of hippocampal neurons from knockouts, NMDA had no neurotoxic effects, determined by lactate dehydrogenase release and nitric oxide synthase (NOS)-dependent cGMP production. Male knockouts and wildtypes (6–8 weeks old) were then injected with NMDA (75 mg/kg; ip) and hippocampal neuronal damages were assessed. Wildtypes developed severe tonic-clonic seizures, whereas knockouts had mild seizure activity (motionless). In knockouts, the NOS activity, caspase-3 expression/activity and the number of TUNEL-positive cells were significantly low. Immunochemical analysis revealed decreased expression levels of the NMDA receptor subunit GluN1 and the postsynaptic density protein PSD-95 in knockouts. Extracellular recording from hippocampal slices showed no Mg^2+^/NMDA-mediated epileptiform events in knockouts. In conclusion, these results show a decrease in NMDA neurotoxicity by NBCn1 deletion. Given that acid extrusion has been known to prevent pH decrease and protect neurons from acid-induced damage, our study presents novel evidence that acid extrusion by NBCn1 stimulates neurotoxicity.

## Introduction

The pH in the brain extracellular space and cerebrospinal fluid is normally maintained at pH 7.3, but it can be substantially low under some pathological conditions^1–4^. Severe decrease in pH causes cells to die as it induces Ca^2+^ influx that promotes multiple signaling cascades involving mitochondria destruction, protease release, and subsequent cell death. On the other hand, a mildly low pH can protect cells from damage as it inhibits numerous proteins responsible for membrane discharge, synaptic transmission and signal transduction^5^. One of these proteins is the N-methyl-D-aspartic acid receptor (NMDAR) that causes cell death upon excessive or prolonged exposure to glutamate^6^. NMDAR has the half maximum inhibition of pH 7.3 close to physiological pH^7^ and this stiff pH dependence has been a model of beneficial effects of mild acidosis^8^. In this regard, studying the mechanism of pH-regulation in neurons is important for advancing our knowledge of glutamate neurotoxicity that occurs in brain insults such as seizures, cerebral ischemia and traumatic brain injury. However, not many reports are presently available for understanding the role of acid/base transporters in these disorders.

The sodium bicarbonate cotransporter NBCn1/SLC4A7 regulates intracellular pH (pH_i_) in neurons^5,9^. NBCn1 is largely found at synapses, particularly in the excitatory postsynaptic membranes^10,11^, where it moves HCO_3_^−^ into postsynaptic neurons. Because HCO_3_^−^ interacts with H^+^ and raises pH_i_, NBCn1 is upregulated under acidic conditions to recover from intracellular acidification^12–14^. Interestingly, in hippocampal neuronal cultures from rats, NBCn1 knockdown reduces glutamate-mediated neurotoxicity^12^. The knockdown attenuates neuronal death caused by glutamate in Mg^2+^-free conditions and the effects are more prominent with time. NBCn1 can cluster with NMDAR via the postsynaptic density protein PSD-95 to form a large protein complex^11,15^. These results suggest that NBCn1 and NMDAR would coordinate together in a manner that NBCn1 upregulation under acidic conditions enhances NMDAR presentation in membranes and as a result cell death increases. Nonetheless, it is currently not shown whether this *in vitro* observation has any functional consequence in the brain.

In this study, we examined NMDA-induced neurotoxicity in NBCn1 knockout (KO) mice to determine whether a similar coordination also occurs in the mouse brain. The experiments were focused on the hippocampus that is highly vulnerable to glutamate neurotoxicity particularly implicated in seizures^16,17^. The results show low or negligible cell death in knockouts, comparable to the *in vitro* results from primary cultures. These mice are also protected from epileptiform-like events mediated by NMDA. The results imply that NBCn1 can be a target for neuroprotection from acidosis-related brain damage.

## Materials and Methods

### Mice

All experiments described in this study were conducted in accordance with the National Institute of Health Guide for the Care and Use of Laboratory Animals. Experimental protocols were approved by the Institutional Animal Care and Use Committee at Emory University.

All experiments in this study were performed with male mice to minimize a potential gender difference. NBCn1 KO mice by Slc4a7 gene targeting with background of C57BL/6J were obtained from Drs. Christian Aalkjaer and Ebbe Boedtkjer (Aarhus University, Denmark). The generation and basic characterization of KO mice were described previously^18^. Heterozygotes were bred to generate KO mice and wildtype (WT) littermates, and genotyping was done by PCR of tail DNA. Mice were housed on a 12 h light/dark cycle and provided with standard chow and water *ad libitum*. Experiments were conducted during the light phase.

### Primary cultures

Primary cultures of hippocampal neurons from postnatal mice were prepared using the protocol^19^ with slight modification. Briefly, brains were removed from P5–P7 postnatal mice after decapitation and placed in sterile ice-cold Hanks’ Balanced Salt solution HBSS (ThermoFisher Scientific, Waltham, MA, USA). Hippocampi were cut out under a dissecting microscope and digested with papain (Worthington Biochemical Corporation; Lakewood, NJ) for 15 min at 37 °C. Tissues were then rinsed out with HBSS with 10% fetal bovine serum and treated with NeuroBasal Medium (NBM) with B-27 supplement (Thermo Fisher Scientific) and titurated until cells were in suspension. Cells were plated onto a poly lysine-coated 24-well plate in NBM/B-27 media and 0.5 mM glutamate. Two days later, media were replaced to remove unattached cells. Neurons were incubated in a 5% CO_2_ humidified chamber at 37 °C for 7–10 days.

### Lactate dehydrogenase (LDH) release

For measurement of LDH release, neurons in a 24-well plate (1 × 10^4^ cells/well) were rinsed twice with exposure media (mM: 144 NaCl, 5 KCl, 2 CaCl_2_, 10 glucose, 10 glycine, 10 HEPES, pH 7.4) and treated with 0–300 μM NMDA in exposure media for 1 h. Media were then rinsed twice and incubated in NBM/B-27 for 24 h. Total LDH release was achieved by breaking neurons with 1% Triton X-100, and the media (no cells) served as a background. LDH release from neurons was quantitated using the Cytotoxicity Detection kit LDH (Roche Applied Science, Penzberg, Germany). The amount of formazan produced in culture supernatants was determined by absorbance at 490 nm. Cell death was expressed as the percentage of the total LDH release after background subtraction.

### Nitric oxide synthase (NOS)

Neurons in a 24-well plate were rinsed three times with exposure media and pretreated with 0 or 100 μM of N-nitro-L-arginine methyl ester (L-NAME) for 30 min. Then, 100 μM of NMDA or none was added and the incubation continued for 15 min. Cells were lysed with 0.1 M HCl after removing the medium and centrifuged at 600 × *g* to collect the supernatants. cGMP levels were measured using a cGMP Enzyme Immunoassay kit (Sigma–Aldrich) according to the manufacturer’s protocol. The measurements of acetylated samples and cGMP standards were made with absorbance at 405 nm.

### Behavioral assessment of seizure activity

Male NBCn1 KO mice and WT mice (6–8 weeks old) were intraperitoneally injected with NMDA (75 mg/kg body weight). Each mouse was administered with one injection and tested separately. Similar to kainic acid, NMDA causes seizures by directly stimulating the glutamatergic system and its manifestation of seizures is distinct from seizures induced by the commonly used pentylenetetrazole (PTZ) that inhibits GABA receptors. Therefore, the severity of seizures in this study was scored using a modified form of the Racine scale suitable for glutamate-induced seizures^20^, in which stage 0 is normal behavior; 1 immobility; 2 forelimb and/or tail extension, giving a rigid posture; 3 automatism such as repetitive scratching, circling or head bobbing; 4 forelimb clonus, rearing and falling; 5 continuous repeats of score 4; 6 severe tonic-clonic seizures; 7 death. Seizures were video recorded. Severity of seizures, latency to onset of convulsive seizures and highest scores were measured over a 25-min observation period.

### Nitric oxide production assay

Hippocampal lysates were prepared from mice 1 hour after injection of NMDA or saline. Nitric oxide (NO) production was determined using fluorimeteric Nitric Oxide Synthase Detection System (Sigma-Aldrich, cat. #: FCANOS1; St Louis, MO, USA) according to the manufacturer’s protocol. Lysates were incubated with the 4,5-diaminofluorescein (DAF) diacetate which converts to DAF and reacts with NO to form triazolo fluorescein. The fluorescent product was quantitated with an excitation filter at 492 nm and an emission filter at 515 nm using a Synergy 4 Microplate Reader (BioTek; Winooski, VT, USA).

### Caspase-3 activity assay

Active caspase-3 was determined in hippocampal lysates prepared from mice 3 days after NMDA injection. Caspase-3 activity was measured using a Caspase-3 Assay Kit (MilliporeSigma, Burlington, MA, USA) according to the manufacturer’s protocol. Lysates were incubated with the substrate Acetate-Asp-Glu-Val-Asp *p*-nitroanilide (Ac-DVED-pNA) at 37 °C overnight and caspase-3 activity was determined by measuring the absorbance of pNA at 405 nm using a Synergy 4 Microplate Reader. Incubation with purified caspase-3 in the presence or absence of the inhibitor Ac-DVED-CHO served as controls.

### TUNEL assay

TUNEL staining in the mouse hippocampus was performed 3 days after NMDA administration using the *In Situ* Cell Death Detection Kit (Roche) according to the manufacturer’s protocol. After fixation in ethanol–acetic acid, brain sections were treated with proteinase K and permeabilized with 0.5% Triton X-100. The sections were then incubated in the TUNEL reaction mixture containing terminal deoxynucleotidyl transferase and nucleotide mixture for 60 min at 37 °C in the dark. The staining was visualized using Converter-POD with 3,3-diaminobenzidine (DAB) supplied with the kit. TUNEL-positive cells were counted per millimeter square on DAB-staining images using ImageJ software (NIH; Bethesda, MD, USA).

### Immunoblot

Immunoblotting of lysates from the mouse hippocampus was performed as described before^11^ with slight modification. The blot was incubated with the anti-caspase-3 antibody (cat. #: 9662; Cell Signaling Technology; Danvers, MA, USA). The immunoreactive bands were visualized with an ECL chemiluminescence kit (GE Healthcare Bio-Sciences; Pittsburgh, PA, USA). The blot was stripped and then reprobed for β-actin. Densitometric analysis of immunoreactive bands was performed using ImageJ. Pixel intensities of caspase-3 were normalized to β-actin after background subtraction. Full length blots and validation controls are shown in the Supplemental Information (Fig. S1).

### Immunohistochemistry

Brains from adult mice were fixed in 4% paraformaldehyde, dehydrated in 30% sucrose, and embedded in OCT compound or paraffin. Thirty micron-thick sections were washed in phosphate-buffered saline PBS containing 1% H_2_O_2_, blocked with 10% normal goat serum at 37 °C for 1 h, and then incubated with a rabbit anti-caspase-3 antibody (1:200) in 3% normal goat serum at 4 °C overnight. After washes, sections were incubated with a biotinylated secondary antibody (1:500; Vector Laboratories, Burlingame, CA, USA) and then with a Vector Elite ABC Kit (Vector Laboratories). Sections were stained with DAB and images were visualized using a Zeiss Axiovert 135 microscope (Oberkochem, Germany) using a Plan Neofluar 16× and 40× lens (numerical aperture 0.75). For GluN1 and PSD-95 immunofluorescence, brain sections were incubated with a mouse anti-NR1 (GluN1) antibody (1:100; cat. #: 556308, BD Biosciences, San Jose, CA, USA), or a mouse anti-PSD-95 antibody (1:100; cat. #: 1596, MilliporeSigma) at 4 °C overnight. Secondary antibodies were Alexa Fluor 594 anti-mouse IgG (cat. #: A-21203; Thermo Fisher) for GluN1 and Alexa Fluor 488 anti-mouse IgG (cat. #: A-11029; Thermo Fisher) for PSD-95. For immunofluorescence of two other neuronal Na/HCO_3_ transporters NCBE and NDCBE, brain paraffin sections (5 μm thick) were incubated with a rabbit anti-NCBE antibody (1:200)^21^ and a rabbit anti-NDCBE antibody(1:100)^22^. The secondary antibody was Alexa Fluor 594. Nuclei were stained with DAPI solution (Thermo Fisher). Images were visualized using an Olympus Fluoview FV1000 confocal microscope with a UPLFLN 40× and 60× lens (numerical aperture 1.3). Quantification of fluorescence was done using the protocol we previously described^11^.

### Extracellular recording of epileptiform-like activity

Hippocampi were removed from postnatal P15–P21 mice and transverse slices of 300 µm in thickness were made using a vibroslicer (Leica; Buffalo Grove, IL, USA) in ice-cold solution containing (in mM): 124 NaCl, 7 MgCl_2_, 3 KCl, 1.25 KH_2_PO_4_, 0.25 CaCl_2_, 26 NaHCO_3_, 1.3 Na-ascorbate, and 10 glucose (saturated with 95% O_2_ and 5% CO_2_). The slices were incubated for at least 1 hour in the above solution before recording in an interface-type chamber perfused with artificial cerebrospinal fluid (ACSF) containing: 124 NaCl, 3 KCl, 2 CaCl_2_, 1 MgCl_2_, 1.25 NaH_2_PO_4_, 26 NaHCO_3_, and 10 glucose, saturated with 95% O_2_/5% CO_2_. The slices were visualized by an upright Ti Eclipse microscope (Nikon; Melville, NY, USA). Recordings were achieved with an Axoclamp 2B amplifier (Molecular Devices; San Jose, CA, USA) in a bridge mode from the CA3 stratum pyramidale using a thick-walled glass micropipette (40–50 MΩ) filled with ACSF. Signals were digitized with a Digidata 1440A (Molecular Devices) and stored in the computer with pClamp 10 (Molecular Devices). Epileptiform-like activity was triggered by removing extracellular Mg^2+^. The time interval between the first and the last spike in each epileptiform-like event was measured as the duration of an event, and the time interval between the onsets of the first population spike in each event was measured as the delay between events. Recordings were also performed with the NMDAR antagonist 2-amino-5-phosphonovaleric acid APV (50 μM), the GABA_A_ receptor antagonist gabazine (10 μM) and the HCO_3_^−^ transporter antagonist 4,4′-diisothiocyano-2,2′-stilbene disulphonic acid DIDS (25–50 μM). Recordings were low-pass filtered at 100 Hz and high-pass filtered at 1 Hz.

### Statistical analysis

Data were reported as mean ± standard error of the mean (SEM). The significance of the difference between means was determined using i) unpaired, two-tailed Student *t*-test when analyzing highest seizure scores, seizure latency, immunofluorescence and epileptiform-like spikes, and ii) two-way ANOVA with Sidak post hoc test when analyzing TUNEL staining and LDH release in primary cultures, caspase-3 activity/expression in the hippocampus and seizure severity over time. Two-way ANOVA with Tukey post hoc test was used when analyzing cGMP production. Degree of freedom (*F*) and *p* value in two-way ANOVA were for genotype × treatment interaction except when indicated, and *p* < 0.05 was considered significant. Analysis was made using Prism 7 (GraphPad Software; La Jolla, CA, USA).

## Results

### NMDA excitotoxicity is inhibited in hippocampal neuronal cultures from NBCn1 KO mice

**Figure 1:**
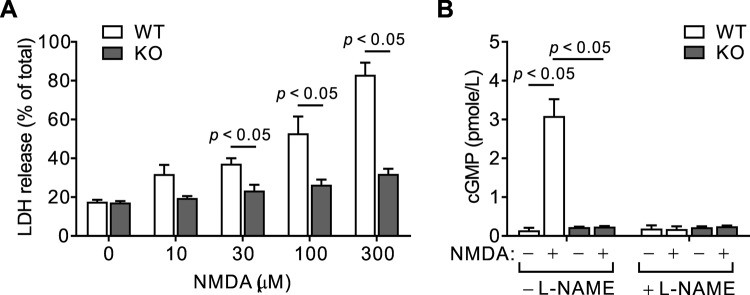
NMDA excitotoxicity is inhibited in hippocampal neuronal cultures from NBCn1 KO mice. (**A**) Lactate dehydrogenase (LDH) release assay. Hippocampal neurons isolated from postnatal mice were exposed to 0–300 μM of NMDA and the amount of LDH released from damaged cells was determined. Total release was achieved by breaking cells with 1% Triton X-100. Cytotoxicity was expressed as a percentage of total LDH release (*n* = 3/group in WT and 6/group in KO). (**B**) cGMP production. Neurons were pretreated with 0 or 100 μM L-NAME for 30 min and then with 0 or 100 μM NMDA for 15 min. Cells were lysed and cGMP levels in the supernatants were quantitated with cGMP standards using an assay kit (*n* = 6/group in WT & 7/group in KO).

As described in the Introduction, NBCn1 knockdown in hippocampal neuronal cultures from rats reduces glutamate-mediated neurotoxicity^12^. To test whether a similar effect occurs in mouse neurons, we cultured hippocampal neurons from mice (P5–P7; 7–10 DIV) and performed NMDA cytotoxicity assays. Figure 1A shows LDH release from WT *vs*. KO neurons at 0–300 μM NMDA. Compared to WT neurons, KO neurons revealed a significantly decreased release (*F*_4,35_ = 15.97, *p* < 0.05 in two-way ANOVA; *n* = 3/group in WT mice & 6/group in KO mice). The difference was more evident at higher doses of NMDA. Figure 1B shows NOS activity assessed by cGMP production in the absence or presence of the NOS antagonist L-NAME. Following 100 μM NMDA treatment, NOS-mediated cGMP production was significantly high in WT neurons, whereas it was minimal in KO neurons (*F*_1,22_ = 52.72, *p* < 0.05 in two-way ANOVA; *n* = 6/group in WT mice & 7/group in KO mice). We also used 300 μM NMDA and found similar results (data not shown). Thus, similar to NBCn1 knockdown in rat hippocampal neurons, NBCn1 knockout in mouse hippocampal neurons inhibits NMDA neurotoxicity.

### NBCn1 KO mice are less vulnerable to NMDA-mediated seizures

**Figure 2:**
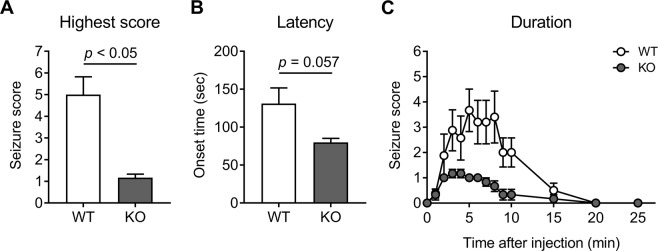
NBCn1 KO mice are less vulnerable to NMDA-mediated seizures. (**A**) Mean highest seizure score. WT and KO mice were injected with NMDA (75 mg/kg; i.p.) and seizure behaviors were scored using a revised Racine scale for glutamate-induced seizure (see Methods). Data were averaged from *n* = 8 WT and 6 KO mice. (**B**) Latency. The time between injection and onset of seizure was calculated. (**C**) Seizure activity over time after NMDA injection. The highest level of seizure activity was scored every min during the first 10 min and then every 5 min for the remaining time. WT mice that reached stage 7 (death) were excluded from the mean value because of the constantly highest scores after death.

To test whether the above *in vitro* observation also occurs in the brain and has any functional consequence, we injected NMDA (75 mg/kg body weight, *i.p*.) into adult mice and determined animals’ seizure behaviors and neurotoxicity in the hippocampus. NMDA is a highly potent convulsant that induces generalized tonic-clonic seizures with short latencies in rodents^23,24^. Consistent with this convulsant effect, NMDA injection revealed a progression of severe seizure behaviors in mice. Assessed by a scale that quantifies seizure severity (see Materials and Methods), the mean highest score was 5.0 ± 0.8 for WT control mice (*n* = 8) and 1.2 ± 0.2 for NBCn1 KO mice (*n* = 6; *p* < 0.05, unpaired two-tailed Student t-test) (Fig. 2A). The latency representing the time between injection and onset of seizure was similar (*p* = 0.057) as shown in Fig. 2B, but we note that the latency can be affected by many factors including different behavioral categories^25^. Figure 2C shows seizure activities over 25 min after NMDA injection. Two-way ANOVA analysis resulted in significantly low seizure activity in KO mice (*F*_14,144_ = 2.22; *p* = 0.009 for interaction of genotype and score), demonstrating a decreased vulnerability to seizures in these mice. Seizures were observed mostly within 10 min after injection and then abolished at 20 min.

### NBCn1 KO mice are protected from the early and mid stages of apoptosis in the hippocampus

**Figure 3:**
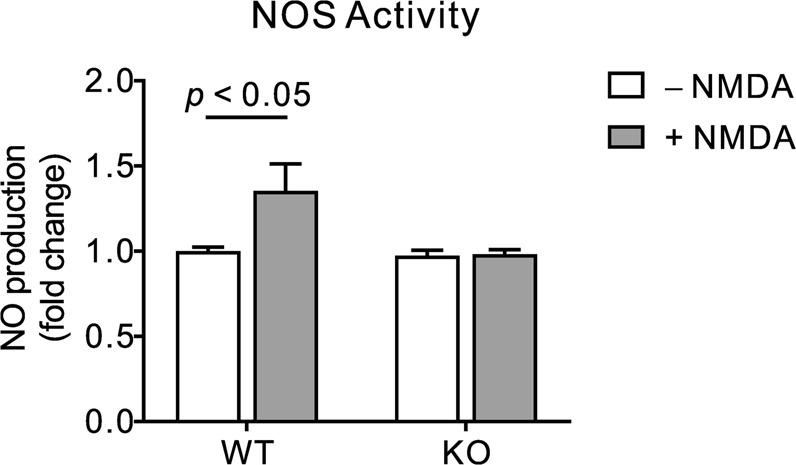
NO production is inhibited in the hippocampus of NBCn1 KO mice. Hippocampal lysates were isolated from WT and KO mice 1 hr after injection with NMDA (*n* = 6/genotype) or saline (*n* = 6/genotype), and NO production was assessed by measuring triazolo fluorescein, a reaction product with NO, using an assay kit. Data were presented as fold change relative to lysates from untreated WT mice.

**Figure 4:**
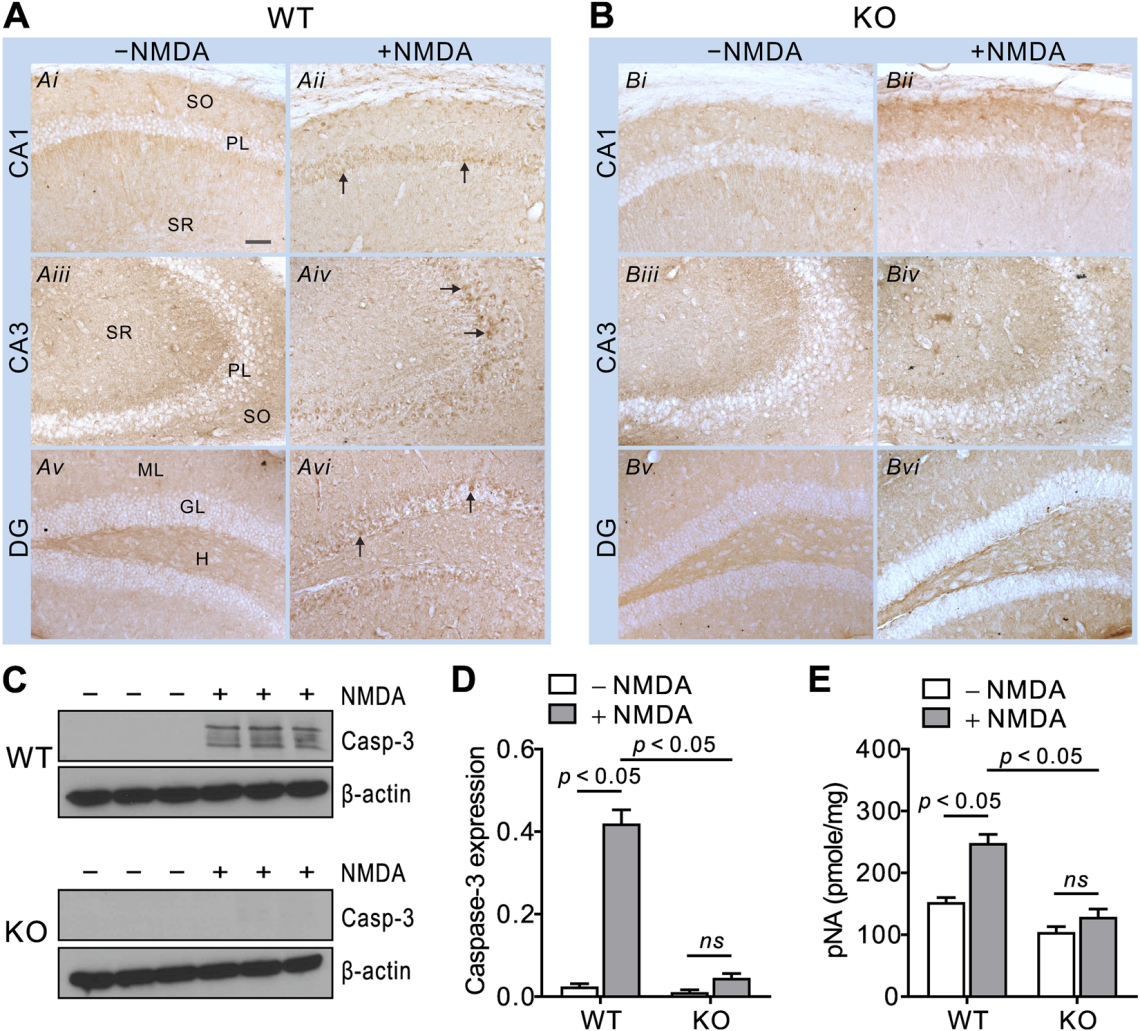
Caspase-3 expression and activity are inhibited in the hippocampus of NBCn1 KO mice. (**A**,**B**) Caspase-3 immunostaining in the hippocampal sections from WT and KO mice 3 days after NMDA injection (*n* = 4/group). Images were taken from pyramidal CA1 and CA3 regions and dentate gyrus (DG). Representative caspase-3-stained cells are shown in *arrows*. SO, stratum oriens; PL, pyramidal layer; SR, stratum radiatum; ML, molecular layer; GL, granular layer; H, hilus. The scale bar is 50 μm and applies to all images. (**C**) Caspase-3 immunoblot. Blots of hippocampal lysates were probed with a caspase-3 antibody, then striped and reprobed with a β-actin antibody. Multiple bands of caspase-3 cleaved from procaspase are shown. Full length blots are available in the Supplemental Information (Fig. S1). (**D**) Quantitation of caspase-3 expression. Pixel intensities of immunoreactive bands were quantitated using ImageJ. Caspase-3 was normalized to β-actin after background subtraction (*n* = 3/group). *ns*, not significant. (**E**) Caspase-3 activity. Hippocampal lysates were prepared from WT and KO mice 3 days after NMDA injection (*n* = 6/group) and assessed for quantitation of *p-*nitroanilide (pNA) hydrolyzed by caspase-3 using an assay kit. The unit is pmole of pNA per mg of total protein. *ns*, not significant.

To examine an early-stage apoptosis induced by NMDA in the hippocampus, we first measured NO production which reflects NOS activation initiated by Ca^2+^ influx via NMDAR^26^. Figure 3 shows the levels of NO production in the hippocampal lysates 1 hour after NMDA injection. The production was significantly increased in WT mice, but not in KO mice (*F*_1, 20_ = 4.39, *p* < 0.05 in two-way ANOVA; *n* = 6/group). Saline injection as a control had no effect. Lack of NO production in response to NMDA is in agreement with reduced endothelial NO production in these mice^18^. We then determined caspase-3 expression, a mid-stage apoptosis event characterized by loss of mitochondrial membrane integrity^27^. Figure 4A,B show images of caspase-3 immunostaining in hippocampal sections from mice 3 days after NMDA or saline injection (*n* =4/group). In WT mice, the number of active caspase-3-positive neurons was increased in the pyramidal layers and dentate gyrus granular layer. Neurons in other areas including stratum oriens, stratum radiatum, and hilus were also stained. However, in KO mice, the staining was insignificant. This differential expression between groups was also assessed by immunoblot (Fig. 4C). Multiple forms of active caspase-3 (largely 17 KDa and 20 KDa cleaved from procaspase) were produced in WT mice. In contrast, active caspase-3 production was absent in KO mice. Quantitating pixel intensities of immunoreactive bands by ImageJ (Fig. 4D) confirmed the significant difference in caspase-3 production between genotypes (*F*_1, 8_ = 101.5, *p* < 0.05 in two-way ANOVA; *n* = 3/group). Furthermore, in the caspase-3 activity assay (Fig. 4E), NMDA injection caused the enzymatic conversion of the substrate Ac-DVED-pNA to pNA to increase in WT mice, but remain at basal levels in KO mice (*F*_1,17_ = 5.12, *p* = 0.037 in two-way ANOVA; *n* = 3/saline group and 7–8/NMDA group). These results demonstrate that NMDA has no effect on caspase-3 expression and activity in NBCn1 KO mice.

### NBCn1 KO mice are protected from the late stage of apoptosis in the hippocampus

**Figure 5:**
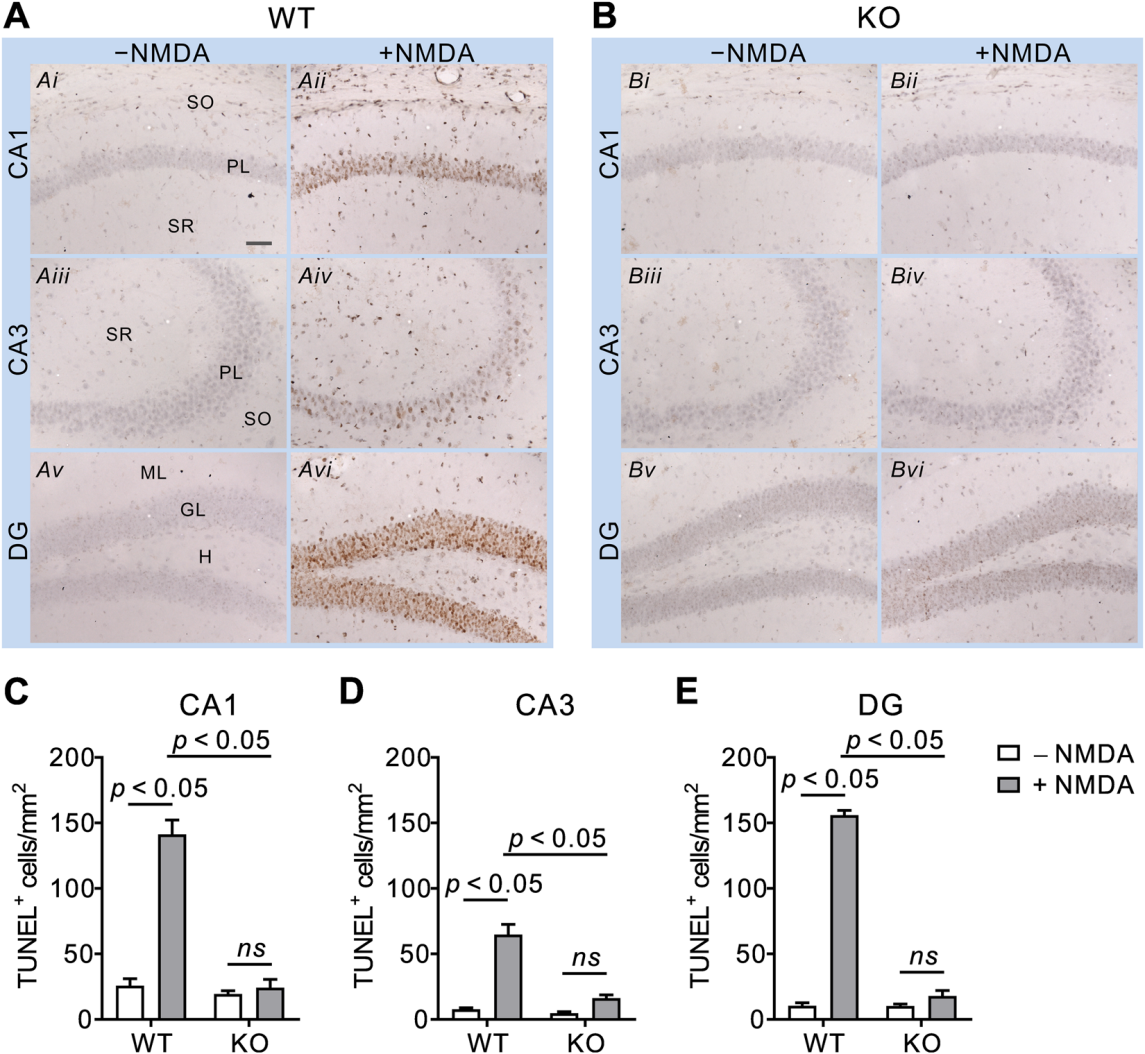
TUNEL-positive cells are low in the hippocampus of NBCn1. (**A**,**B**) TUNEL staining was performed with the hippocampus of WT mice and KO mice 3 days after NMDA injection (*n* = 4/group). Representative images were taken from CA1 (*i* & *ii*), CA3 (*iii* & *iv*) and DG (*v* & *vi*) regions. The abbreviations for areas are described in Fig. 4. The scale bar is 50 μm and applies to all images. (**C**–**E**) Quantitation of TUNEL-positive cells. TUNEL-positive cells in a total field area per slide were counted using the particle analysis function in ImageJ software. Results were expressed as TUNEL^+^ cell number per mm^2^ (*n* = 4/group). Bar legends in E are for all graphs. *ns*, not significant.

Next, we performed a TUNEL assay that determines DNA fragmentation associated with cellular structural changes, a hallmark of late-stage apoptosis. A large number of TUNEL-positive cells were observed in the hippocampal sections from WT mice after NMDA injection (*n* = 4/group; Fig. 5A). These cells were found in most areas with prominent staining in the pyramidal layer and dentate gyrus granular layer. In contrast, the number of TUNEL-positive cells was rarely detectable in KO mice after injection (Fig. 5B). Most cells in these mice were unstained or weakly stained. Quantitation of TUNEL-positive cells confirmed this difference between genotypes (*p* < 0.05 for all regions in two-way ANOVA; *n* = 4/group), as shown in Fig. 5C–E. Together with the above data of NO production and caspase-3 expression/activity, the TUNEL assay demonstrates that loss of NBCn1 protects hippocampal neurons from apoptosis in the brain.

### NBCn1 KO mice are protected due to GluN1 downregulation and no Mg^2+^/NMDA-mediated epileptiform activity

**Figure 6:**
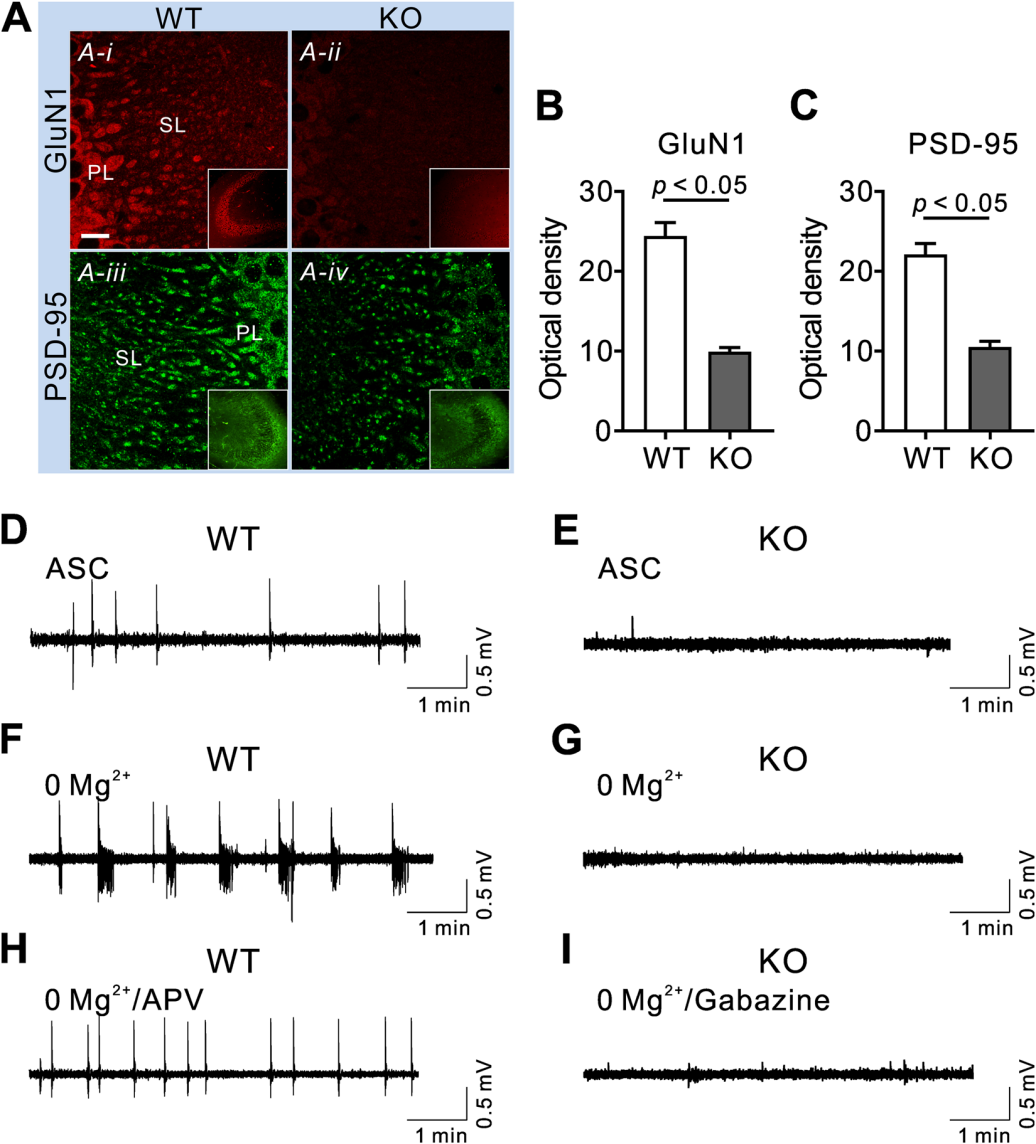
NBCn1 KO mice are protected due to GluN1 downregulation and lack of Mg^2+^/NMDA-mediated epileptiform activity. (**A**) Hippocampal sections containing CA3 regions were labeled with GluN1 and PSD-95 antibodies and immunofluorescence was achieved with Alexa Fluor 594 for GluN1 and Alexa Fluor 488 for PSD-95 (*n* = 4/group). Low-magnification images are shown in insets. PL, pyramidal layer; SL, stratum lucidum. The scale bar is 10 μm and applies to all images. (**B**,**C**) Quantitation of GluN1 and PSD-95. Immunofluorescence in the SL was quantitated using ImageJ (from 20 randomly selected areas in the SL). (**D,E**) Extracellular recordings in the CA3 region showing spontaneous action potentials in the standard ACSF. Compared to WT neurons, KO neurons showed a marked decrease in action potential frequency and amplitude (*n* = 5 WT & 4 KO slices). (**F**,**G**) Epileptiform-like activity. Repetitive membrane discharges with >2 sec duration and ~1 event/min frequency, characteristic of ictal-like events, were triggered by removing extracellular Mg^2+^ (*n* = 7/group). (**H**,**I**) Recordings in 0 Mg^2+^ ACSF in the presence of 50 μM APV (*n* = 7) or 10 μM gabazine (*n* = 3).

The above results of low seizure activity and negligible neurotoxicity in NMDA-injected KO mice led to the question of what caused such changes. Given our previous finding of NBCn1 being capable of clustering with NMDAR via PSD-95^11,15^, we speculated that NBCn1 deletion would alter NMDAR expression in membranes. This possibility was tested by immunohistochemistry of GluN1 and PSD-95 (Fig. 6A). Experiments were focused on CA3 pyramidal neurons, where NBCn1 is highly abundant and its interaction with PSD-95 has been demonstrated^11^. In WT mice, GluN1 immunofluorescence was strong in the pyramidal layer cell bodies and proximal dendrites in the stratum lucidum, consistent with the previous reports^28^. However, in KO mice, GluN1 immunofluorescence was markedly low. The difference between WT and KO mice was evident in both cell bodies and dendrites. Similarly, PSD-95 immunofluorescence was decreased particularly in the stratum lucidum in KO mice. Quantitation of GluN1 and PSD-95 immunofluorescence confirmed their downregulation in KO mice (*p* < 0.05 for both proteins in unpaired, two-tailed Student t-test; *n* = 4/group), as shown in Fig. 6B,C.

To test whether loss of NBCn1 is responsible for decreased seizure activity in mice, we performed extracellular recording of action potentials in CA3 pyramidal neurons in hippocampal slices. In the standard perfusion solution ACSF, WT neurons produced spontaneous action potentials (Fig. 6D), common features of synaptic activity in the neuronal network^29^. KO neurons also produced spontaneous action potentials but with much lower frequency and amplitude (Fig. 6E). The frequency was 0.31 ± 0.01 spike/min for WT neurons (*n* = 5) and 0.07 ± 0.01 spike/min for KO neurons (*n* = 4). Epileptiform-like repetitive membrane discharges were triggered by depleting bath Mg^2+^ in WT neurons, as shown in Fig. 6F. The average event duration (i.e., the time interval between the first and the last spike in each event) in this recording was 16.5 ± 2.3 sec (*n* = 8 events) and the delay between events (i.e., the time interval between the onsets of the first population spikes in two consecutive events) was 58.2 ± 4.1 sec (*n* = 6 events). These are consistent with ictal (seizure)-like events that are characterized with >2 sec duration and about 1 event/min frequency^30,31^. The initial sustained phase and subsequent intermittent population discharges were also consistent with ictal-like events. In KO mice, no epileptiform-like activity was produced after Mg^2+^ depletion (*n* = 7; Fig. 6G). This lack of repetitive spikes was not due to an inability to depolarize cell membranes because KO neurons produced spontaneous action potentials. Figure 6H shows the inhibition of epileptiform-like events by the NMDAR antagonist APV at 50 μM in WT neurons (*n* = 5). Conversely, the GABA_A_ receptor blocker gabazine had no effect in KO neurons (*n* = 3; Fig. 6I). Thus, CA3 neurons in KO mice had no NMDA-mediated epileptiform-like activity, comparable to decreased seizure activity in these animals.

### NCBE and NDCBE expression levels are not decreased in hippocampal neurons of NBCn1 KO mice

**Figure 7:**
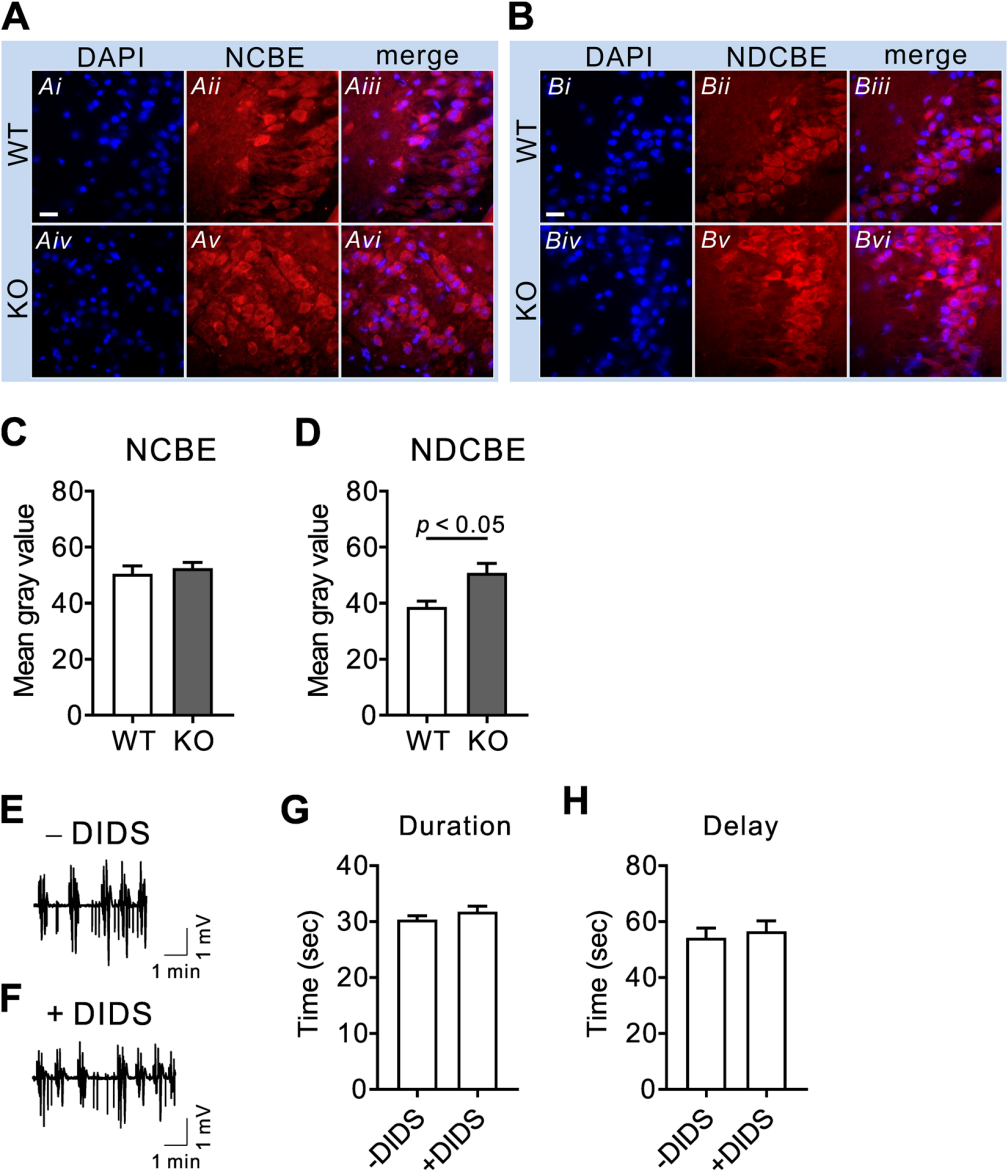
NCBE and NDCBE expression levels are not decreased in hippocampal neurons of NBCn1 KO mice. (**A**,**B**) NCBE and NDCBE immunofluorescence in CA3 regions. Mouse hippocampal slices containing pyramical CA3 regions were labeled with antibodies to the C-terminal ends of NCBE and NDCBE. Nuclei were stained with DAPI. The scale bar in *Ai* applies to all images. (**C**,**D**) Quantitation of NCBE and NDCBE immunofluorescence. Quantitative analysis was performed on cell bodies (*n* = 12–16 fluorescence/group). (**E**,**F**) Epileptiform-like events induced by 0 Mg^2+^ in the absence and presence of 50 μM DIDS (*n* = 3 for each). Bath solutions contained 10 μM gabazine to inhibit Cl^−^ movement via GABA_A_ receptors. (**G**) Event duration. The duration is the time interval between the first and the last spike in each event. (*n* = 36 events). (**H**) Delay between events. The delay between events is the time interval between onset of the first population spikes in two consecutive events (*n* = 36 events).

The above results show evident effect of NBCn1 deletion on epileptiform-like activity; nonetheless, we cannot exclude a possibility that other Na/HCO_3_ transporters in neurons are involved. Both NCBE KO mice^32^ and NDCBE KO mice^33^ show decreased seizure activity, implying that negligible seizure/epileptiform activity in NBCn1 KO mice may occur if NCBE and NDCBE are downregulated. To address this possibility, we determined NCBE and NDCBE expression levels in NBCn1 KO mice by immunohistochemistry. In hippocampal slices containing CA3 neurons, the immunofluorescence of these two transporters was prominent in cell bodies and dendrites (Fig. 7A,B), consistent with the previous reports by Chen *et al*.^21,22^. NCBE immunofluorescence remained similar between WT and KO mice, whereas NDCBE immunofluorescence was increased in KO mice (*p* < 0.05; Student *t*-test; *n* = 3/group; Fig. 7C,D). The increase in NDCBE expression levels, which probably occurred in a compensatory process to recover acidic intracellular pH, is opposite to the predicted decrease. In other experiments, we evaluated the effect of the stilbene derivative DIDS on epileptiform-like activity. Except NBCn1, most Na/HCO_3_ transporters are sensitive to DIDS^34^. Figure 7E shows epileptiform-like events triggered by 0 mM Mg^2+^ in WT hippocampal slices (*n* = 3). The recording was achieved in the presence of gabazine to block Cl^−^ movement via GABA_A_ receptors that may complicate NCBE or NDCBE. Under this condition, DIDS (50 μM) had negligible effect on the epileptiform-like events (Fig. 7F). Averages in event duration and delay between events were similar between slices with and without treatment (*p* > 0.05 for each; *n* = 36 events for each group; Fig. 7G,H).

## Discussion

In this study, we found that NBCn1 KO mice were less vulnerable to NMDA-induced seizure and neurotoxicity in the hippocampus. Furthermore, *in vitro* experiments with primary cultures of hippocampal neurons also showed negligible NMDAR/NOS cell death, consistent with the previous finding of reduced glutamate excitotoxicity by NBCn1 knockdown in hippocampal neuronal cultures from rats^11^. The reason is NMDAR downregulation, as shown by decreased GluN1 expression and lack of epileptiform-like activity in CA3 neurons. Our study provides *in vivo* and *in vitro* evidence that NBCn1 contributes to the progression of glutamate-induced hippocampal damage.

Animal studies show that genetic disruption of SLC4A HCO_3_^−^ transporters leads to changes in seizure vulnerability^32,35–37^. Mice with the gene ablation of acid-loading AE3 (*Slc4a3*^−/−^) develop a reduced seizure threshold in the seizure paradigms of PTZ, pilocarpine, hyperthermia^35^. Neurons from these mice have an elevated pH_i_, which causes neuronal hyperexcitability. In humans, a mutation in AE3 is associated with idiopathic generalized epilepsy^38^. Conversely, the gene ablation of acid-extruding HCO_3_^–^ transporters results in the opposite effects^32,33^. NCBE KO mice (*Slc4a10*^−/−^) develop decreased seizure incidence and mortality in response to PTZ, pilocarpine, and hyperthermia^39^. The latency to onset of seizure is also increased. NDCBE KO mice (*Slc4a8*^−/−^) develop decreased seizure activity in the similar seizure paradigms^33^. The anticonvulsant response is due to impaired release of glutamate vesicles, the pH of which is regulated by NDCBE. Consistent with these reports, our study shows reduced seizure activity in NBCn1 KO mice in response to NMDA. Our finding thus supports the current concept that disrupting an acid extrusion process in neurons reduces a seizure threshold whereas disrupting an acid loading process has an opposite effect. Nonetheless, we note that chromosomal translocation involving the *SLC4A10* gene in humans causes complex partial epilepsy and cognitive dysfunction^40^. As for non-neuronal NBCe2, the results are complicated. Kao *et al*.^41^ reported that NBCe2 KO mice (*Slc4a5*^−/−^) display decreased intracerebral volume and pressure due to abnormal electrolytes in the cerebrospinal fluid and are more resistant to seizure induction by PTZ. On the other hand, Christensen *et al*.^42^ reported that another line of NBCe2 KO mice are not protected from seizure in the PTZ and hyperthermia model although the time lag before seizure development in the heat‐treated hyperventilating mice tends to be longer.

In accordance with reduced seizure activity, NBCn1 KO mice have negligible NMDA neurotoxicity. Boedtkjer *et al*.^18^ reported that NBCn1 KO mice develop reduced NO production and rho-kinase-dependent Ca^2+^ sensitivity in middle cerebral arteries and their ability to regulate cerebral blood flow is hampered. We do not think that the change in cerebral blood flow is the cause for the resistance to NMDA neurotoxicity because the same resistance is also observed in primary cultures. Instead, out data demonstrate that the cause is NMDAR downregulation. The downregulation can be accounted for by failure to form a macromolecular protein complex constituted of NBCn1, PSD-95, and NMDAR. The deletion of NBCn1 in knockouts would cause PSD-95 to be downregulated or misplaced in dendrites. PSD-95 inhibits NMDAR internalization^43^ and thus it is possible that PSD-95 downregulation or misplacement resulted in aberrant expression and/or downregulation of NMDAR. If so, NBCn1 serves to stabilize its binding and clustering partners in membranes. The stabilizing effect of NBCn1 is observed in the synaptic terminals of both retinal photoreceptors and inner ear hair cells, where NBCn1 interacts with other membraneous and cytosolic proteins^44^. The absence of NBCn1 disrupts its protein network, resulting in defects in hearing and/or vision^44^.

An alternative cause of NMDAR downregulation is a pH change in neurons. The mean steady-state pH_i_ in hippocampal neurons from KO mice is 0.1 pH unit lower than that for neurons from WT mice^45^. Despite such a small acidification, it is possible that a prolonged exposure to a mildly acidic pH environment has influenced factors responsible for NMDAR stability in plasma membrane during mouse development. For example, intracellular acidosis leads to extracellular acidosis^3^, which activates Ca^2+^/calmodulin-dependent protein kinase II to phosphorylate NMDAR^46^, resulting in receptor downregulation. Nonetheless, it is difficult to envision how a mildly acidic pH results in NMDAR downregulation, particularly given that the receptors are inhibited under such conditions.

Our study provides a new perspective of pH pathophysiology in the nervous system. Acid extrusion has been considered to prevent severe pH_i_ decrease and protect neurons from acid-induced damage. Our study challenges this view and presents the opposite idea that acid extrusion stimulates neurotoxicity. In particular, we envision that NBCn1 plays a key role in this process. Acidification following seizures will trigger NBCn1 upregulation, which stimulates NMDAR expression in membrane and allows more Ca^2+^ to enter, thus enhancing the cascading apoptotic events that render neurons more vulnerable to neurotoxicity. In our study, NBCn1 facilitates the insult progress in seizure. This idea seeks to shift the current paradigm that the main function of NBCn1 is to recover from acidification that triggers mitochondria destruction, protease release, and subsequent cell death. An interesting question is to what extent NBCn1 affects NMDAR. Na^+^-driven Cl/HCO_3_ exchange (i.e., NDCBE and possibly NCBE) has been recognized as the major transport mechanism in most neurons, but Cl^−^-independent Na/HCO_3_ cotransport (i.e., NBCn1) also shows substantial acid extrusion^1,34,47^. Each transporter has its unique subcellular localization in addition to cell bodies: NDCBE in presynaptic terminals where it regulates pH in synaptic vesicles^48^; NCBE in dendrites in most neurons^22^; and NBCn1 in postsynaptic membranes^49^. NDCBE and NCBE do not cluster with NMDAR, whereas NBCn1 does. Thus, the effects of NBCn1 on NMDAR downregulation should be the largest among three transporters.

In summary, our study recognizes NBCn1 as the protein that not only plays a role in pH_i_ maintenance but also affects NMDAR expression and activity. Our study supports the potential of NBCn1 to serve as a new neuroprotective target for glutamate-induced brain damage, possibly hippocampal damage during the acute and chronic stages of diseases such as hippocampal sclerosis (Ammon’s horn sclerosis), the most common type of neuronal damage in individuals suffering from temporal lobe epilepsy^50,51^. NBCn1 inhibition might be more effective to reduce seizure than the current inhibition of carbonic anhydrase, which adversely causes severe metabolic acidosis^52^. It will be interesting to test whether a blockage of the NBCn1/NMDAR protein complex reduces glutamate neurotoxicity in future experiments.

## Supplementary information

Supplementary Information

## References

[1] Ruffin, V. A., Salameh, A. I., Boron, W. F. & Parker, M. D. Intracellular pH regulation by acid-base transporters in mammalian neurons. *Frontiers in physiology* **5**, http://www.ncbi.nlm.nih.gov/pubmed/24592239 (2014). 10.3389/fphys.2014.00043 PMC3923155 24592239

[2] Katsura, K. & Siesjo, B. K. In *pH and Brain Function* (eds Kaila, K. & Ransom, B.R.) 563–582 (Wiley-Liss, Inc, 1998).

[3] Bevensee, M. O., Alper, S. L., Aronson, P. S. & Boron, W. F. In *The Kidney, Physiology and Pathophysiology* (eds Seldin, D.W. & Giebisch, G.) 391–442 (Raven, 2000).

[4] Lipton P (1999). Ischemic cell death in brain neurons. Physiol Rev..

[5] Chesler M (2003). Regulation and modulation of pH in the brain. Physiological reviews.

[6] Dingledine R, Borges K, Bowie D, Traynelis SF (1999). The glutamate receptor ion channels. Pharmacol.Rev..

[7] Traynelis, S. F. In *pH and Brain Function* (eds Kaila, K. & Ransom, B.R.) Ch. 417–446, (Wiley-Liss, Inc., 1998).

[8] Lam TI (2013). Intracellular pH reduction prevents excitotoxic and ischemic neuronal death by inhibiting NADPH oxidase. Proc Natl Acad Sci USA.

[9] Thornell IM, Bevensee MO (2015). Regulators of Slc4 bicarbonate transporter activity. Frontiers in physiology.

[10] Lee S (2010). Sodium-bicarbonate cotransporter NBCn1 in the kidney medullary thick ascending limb cell line is upregulated under acidic conditions and enhances ammonium transport. Exp.Physiol.

[11] Park HJ (2010). Neuronal expression of sodium/bicarbonate cotransporter NBCn1 (SLC4A7) and its response to chronic metabolic acidosis. Am J Physiol Cell Physiol.

[12] Cooper DS (2009). Sodium/bicarbonate cotransporter NBCn1/slc4a7 increases cytotoxicity in magnesium depletion in primary cultures of hippocampal neurons. Eur.J.Neurosci..

[13] Kwon TH (2002). Chronic metabolic acidosis upregulates rat kidney Na-HCO_3_ cotransporters NBCn1 and NBC3 but not NBC1. Am.J.Physiol Renal Physiol.

[14] Odgaard E (2004). Basolateral Na^+^-dependent HCO_3_^−^ transporter NBCn1-mediated HCO_3_^−^ influx in rat medullary thick ascending limb. J.Physiol.

[15] Lee Hye Jeong, Kwon Min Hyung, Lee Soojung, Hall Randy A., Yun C. Chris, Choi Inyeong (2014). Systematic family-wide analysis of sodium bicarbonate cotransporter NBCn1/SLC4A7 interactions with PDZ scaffold proteins. Physiological Reports.

[16] Barker-Haliski M, White HS (2015). Glutamatergic Mechanisms Associated with Seizures and Epilepsy. Cold Spring Harb Perspect Med.

[17] Dingledine R, Varvel NH, Dudek FE (2014). When and how do seizures kill neurons, and is cell death relevant to epileptogenesis?. Adv Exp Med Biol.

[18] Boedtkjer E (2011). Disruption of Na^+^, HCO_3_^−^ cotransporter NBCn1 (slc4a7) inhibits NO-mediated vasorelaxation, smooth muscle Ca^2+^ sensitivity, and hypertension development in mice. Circulation.

[19] Kaech S, Banker G (2006). Culturing hippocampal neurons. Nature protocols.

[20] Morrison RS (1996). Loss of the p53 tumor suppressor gene protects neurons from kainate-induced cell death. J.Neurosci..

[21] Chen LM (2008). Use of a new polyclonal antibody to study the distribution and glycosylation of the sodium-coupled bicarbonate transporter NCBE in rodent brain. Neuroscience.

[22] Chen LM (2008). Expression and localization of Na-driven Cl-HCO(3)(-) exchanger (SLC4A8) in rodent CNS. Neuroscience.

[23] Velisek, L. In *Models of seizures and epilepsy* Vol. 1 (ed. Schwartzkroin, A., Pitkanen, P. A. & Moshe, S. L.) 127–152 (Elsvier, 2006).

[24] Mares P, Velisek L (1992). N-methyl-D-aspartate (NMDA)-induced seizures in developing rats. Brain Res Dev Brain Res.

[25] Luttjohann A, Fabene PF, van Luijtelaar G (2009). A revised Racine’s scale for PTZ-induced seizures in rats. Physiol Behav.

[26] Traynelis SF (2010). Glutamate receptor ion channels: structure, regulation, and function. Pharmacological reviews.

[27] Janicke RU, Sprengart ML, Wati MR, Porter AG (1998). Caspase-3 is required for DNA fragmentation and morphological changes associated with apoptosis. The Journal of biological chemistry.

[28] Thompson CL, Drewery DL, Atkins HD, Stephenson FA, Chazot PL (2002). Immunohistochemical localization of N-methyl-D-aspartate receptor subunits in the adult murine hippocampal formation: evidence for a unique role of the NR2D subunit. Brain research. Molecular brain research.

[29] Siebler M, Koller H, Stichel CC, Muller HW, Freund HJ (1993). Spontaneous activity and recurrent inhibition in cultured hippocampal networks. Synapse.

[30] McCormick DA, Contreras D (2001). On the cellular and network bases of epileptic seizures. Annu Rev Physiol.

[31] Avoli M (2002). Network and pharmacological mechanisms leading to epileptiform synchronization in the limbic system *in vitro*. Progress in neurobiology.

[32] Jacobs S (2008). Mice with targeted Slc4a10 gene disruption have small brain ventricles and show reduced neuronal excitability. Proc.Natl.Acad.Sci.USA.

[33] Sinning A (2011). Synaptic glutamate release is modulated by the Na+ -driven Cl-/HCO exchanger Slc4a8. J.Neurosci..

[34] Aalkjaer C, Boedtkjer E, Choi I, Lee S (2014). Cation-coupled bicarbonate transporters. Comprehensive Physiology.

[35] Hentschke M (2006). Mice with a targeted disruption of the Cl-/HCO3- exchanger AE3 display a reduced seizure threshold. Mol.Cell Biol.

[36] Parker MD (2018). Mouse models of SLC4-linked disorders of HCO3(-)-transporter dysfunction. Am J Physiol Cell Physiol.

[37] Sinning A, Hubner CA (2013). Minireview: pH and synaptic transmission. FEBS Lett.

[38] Sander T (2002). Association of the 867Asp variant of the human anion exchanger 3 gene with common subtypes of idiopathic generalized epilepsy. Epilepsy research.

[39] Gurnett CA (2008). Disruption of sodium bicarbonate transporter SLC4A10 in a patient with complex partial epilepsy and mental retardation. Arch Neurol..

[40] Krepischi AC (2010). Two distinct regions in 2q24.2-q24.3 associated with idiopathic epilepsy. Epilepsia.

[41] Kao L (2011). Severe neurologic impairment in mice with targeted disruption of the electrogenic sodium bicarbonate cotransporter NBCe2 (Slc4a5 gene). J.Biol.Chem..

[42] Christensen HL (2018). The choroid plexus sodium-bicarbonate cotransporter NBCe2 regulates mouse cerebrospinal fluid pH. The Journal of physiology.

[43] Roche KW (2001). Molecular determinants of NMDA receptor internalization. Nat Neurosci.

[44] Reiners J (2005). Scaffold protein harmonin (USH1C) provides molecular links between Usher syndrome type 1 and type 2. Hum.Mol.Genet..

[45] Schank, J. R. *et al*. Sodium bicarbonate transporter NBCn1 modulates alcohol consumption and alcohol-induced behaviors in mice Addiction biology, submitted (in revision) (2019).

[46] Chung HJ, Huang YH, Lau LF, Huganir RL (2004). Regulation of the NMDA receptor complex and trafficking by activity-dependent phosphorylation of the NR2B subunit PDZ ligand. The Journal of neuroscience: the official journal of the Society for Neuroscience.

[47] Liu Y, Yang J, Chen LM (2015). Structure and Function of SLC4 Family [Formula: see text] Transporters. Frontiers in physiology.

[48] Burette AC (2012). The sodium-driven chloride/bicarbonate exchanger in presynaptic terminals. J.Comp Neurol..

[49] Lee S (2012). PSD-95 Interacts with NBCn1 and Enhances Channel-like Activity without Affecting Na/HCO_3_ Cotransport. Cell Physiol Biochem.

[50] Blumcke I, Thom M, Wiestler OD (2002). Ammon’s horn sclerosis: a maldevelopmental disorder associated with temporal lobe epilepsy. Brain Pathol.

[51] Mathern GW (1997). Human hippocampal AMPA and NMDA mRNA levels in temporal lobe epilepsy patients. Brain.

[52] Leniger T, Thone J, Wiemann M (2004). Topiramate modulates pH of hippocampal CA3 neurons by combined effects on carbonic anhydrase and Cl-/HCO_3_ exchange. Br.J.Pharmacol..

