# Supplementary material for: Deletion of the Na/HCO3 Transporter NBCn1 Protects Hippocampal Neurons from NMDA-induced Seizures and Neurotoxicity in Mice
Source: Sci Rep. 2019 Nov 5;9:15981. doi: 10.1038/s41598-019-52413-0 (PMC6831677; doi:10.1038/s41598-019-52413-0)
Supplement: Supplementary file 1 — Supplementary Information [file 41598_2019_52413_MOESM1_ESM.pdf]

Deletion of the Na/HCO_3 Transporter NBCn1 Protects Hippocampal Neurons from NMDA-induced Seizures and Neurotoxicity in Mice

Hae Jeong Park[1], Carlos E. Gonzalez-Islas[2,3], Yunhee Kang[4], Jun Ming Li[3], and Inyeong Choi[3,*]

[1]Department of Pharmacology, Kyung Hee University School of Medicine, Seoul, South Korea

[2]Doctorado en Ciencias Biologicas, Universidad Autonoma de Tlaxcala, Tlax, Mexico

[3]Department of Physiology and [4]Department of Human Genetics, Emory University School of Medicine, Atlanta, GA 30322, USA

*Corresponding author: Inyeong Choi, Department of Physiology, Emory University School of Medicine, 605 Whitehead Research Building, 615 Michael Street, Atlanta, GA 30322, USA. Phone: 404-712-2092, FAX: 404-727-2648, Email: ichoi@emory.edu

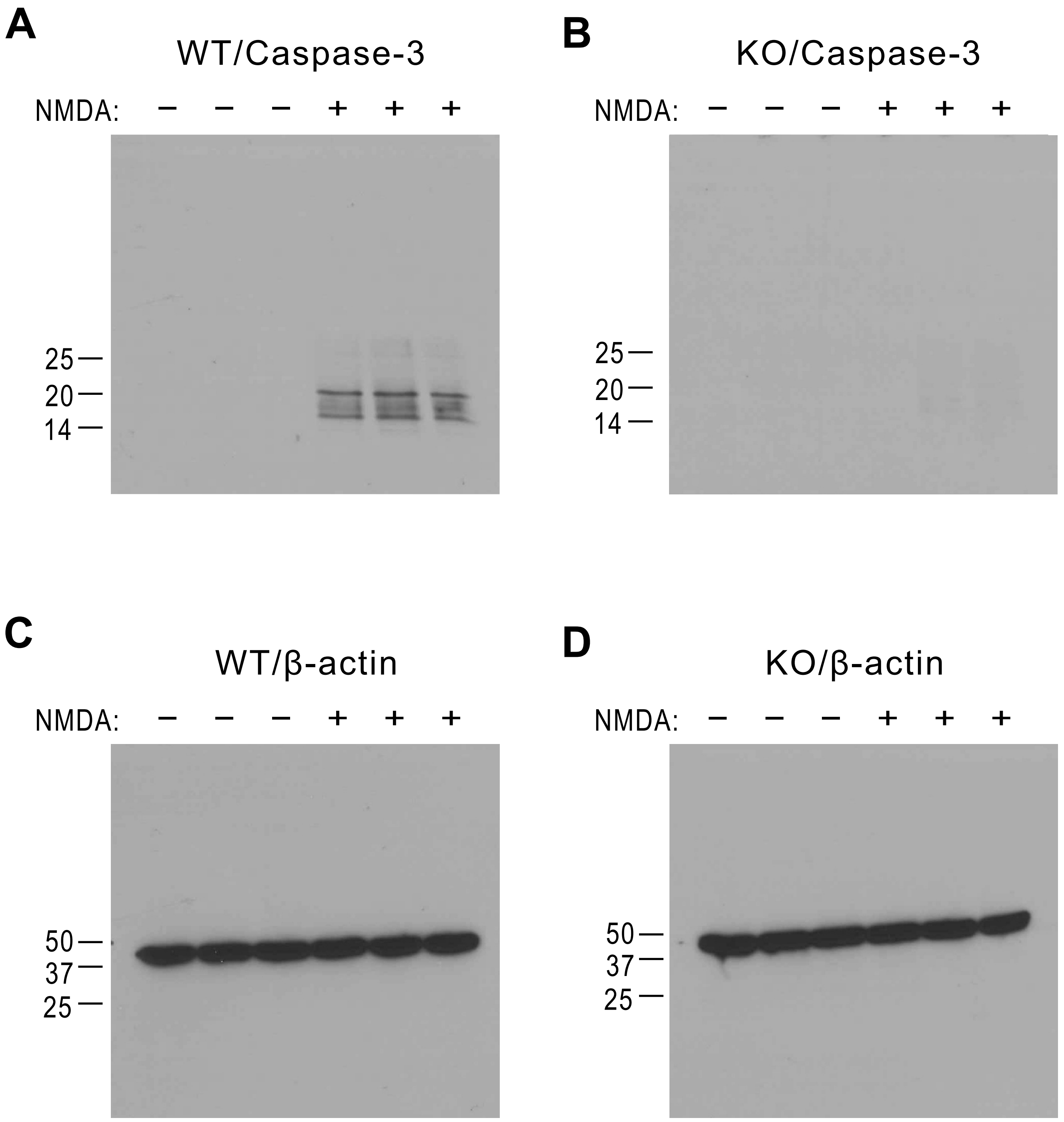

Supplementary Figure S1. Full-length blots of caspase-3 and β-actin. Immunoblots were performed with crude plasma membranes of hippocampal lysates from WT vs. KO mice after injection of saline (–) or NMDA (+). Blots were probed with caspase-3 antibody (**A**, **B**), and then striped and reprobed with β-actin antibody (**C**, **D**). Immunoreactive bands were detected using a chemiluminescence detection system. The molecular weight markers are in kilodaltons (kDa).
